# Genome-wide algorithm for detecting CNV associations with diseases

**DOI:** 10.1186/1471-2105-12-331

**Published:** 2011-08-09

**Authors:** Yaji Xu, Bo Peng, Yunxin Fu, Christopher I Amos

**Affiliations:** 1 Department of Epidemiology, The University of Texas MD Anderson Cancer Center, 1155 Pressler St., Houston, Texas 77030, USA; 2 Division of Biostatistics, The University of Texas School of Public Health, 1200 Pressler St., Houston, Texas 77030, USA; 3 Department of Epidemiology and Public Health, Yale University School of Medicine, 60 College St., New Haven, Connecticut 06520, USA

## Abstract

**Background:**

SNP genotyping arrays have been developed to characterize single-nucleotide polymorphisms (SNPs) and DNA copy number variations (CNVs). Nonparametric and model-based statistical algorithms have been developed to detect CNVs from SNP data using the marker intensities. However, these algorithms lack specificity to detect small CNVs owing to the high false positive rate when calling CNVs based on the intensity values. Therefore, the resulting association tests lack power even if the CNVs affecting disease risk are common. An alternative procedure called PennCNV uses information from both the marker intensities as well as the genotypes and therefore has increased sensitivity.

**Results:**

By using the hidden Markov model (HMM) implemented in PennCNV to derive the probabilities of different copy number states which we subsequently used in a logistic regression model, we developed a new genome-wide algorithm to detect CNV associations with diseases. We compared this new method with association test applied to the most probable copy number state for each individual that is provided by PennCNV after it performs an initial HMM analysis followed by application of the Viterbi algorithm, which removes information about copy number probabilities. In one of our simulation studies, we showed that for large CNVs (number of SNPs ≥ 10), the association tests based on PennCNV calls gave more significant results, but the new algorithm retained high power. For small CNVs (number of SNPs *<*10), the logistic algorithm provided smaller average p-values (e.g., *p *= 7.54*e *- 17 when relative risk *RR *= 3.0) in all the scenarios and could capture signals that PennCNV did not (e.g., *p *= 0.020 when *RR *= 3.0). From a second set of simulations, we showed that the new algorithm is more powerful in detecting disease associations with small CNVs (number of SNPs ranging from 3 to 5) under different penetrance models (e.g., when *RR *= 3.0, for relatively weak signals, *power *= 0.8030 comparing to 0.2879 obtained from the association tests based on PennCNV calls). The new method was implemented in software GWCNV. It is freely available at http://gwcnv.sourceforge.net, distributed under a GPL license.

**Conclusions:**

We conclude that the new algorithm is more sensitive and can be more powerful in detecting CNV associations with diseases than the existing HMM algorithm, especially when the CNV association signal is weak and a limited number of SNPs are located in the CNV.

## Background

Single-nucleotide polymorphisms (SNPs), variable number of tandem repeats (VNTRs) (e.g., mini- and microsatellites), presence or absence of transposable elements (e.g., Alu elements), and structural alterations (e.g., deletions, duplications, and inversions) are the common forms of genomic variability [1]. Ranging from one kilobase to several megabases, copy number variations (CNVs) are segments of DNA that differ in copy numbers when two or more genomes are compared [2]. Originally, the definition of CNV was borrowed from the concept of segmental duplication that was arbitrarily defined as 1 kb in length. Actual CNV size may be far smaller than those defined in HapMap samples [3]. As the technologies have developed, CNVs no longer need to be greater than 1 kb to be detectable [4]. One may study CNVs at a single nucleotide level if the false positive rate in a CNV calling algorithm can be controlled to a low level. CNVs may be inherited, but they can also be produced by de novo mutations [5]. In 2006, a total of 1,447 copy number variable regions (CNVRs) were identified through a study of 270 HapMap samples from four populations using SNP genotyping platforms and clone-based comparative genomic hybridization technologies, and these were estimated to affect 12% of the genome [6]. In a more recent study using a specialized and sensitive technique called fosmid cloning, 1,695 sites of structural variation (including 747 deletions, 724 insertions and 224 inversions) were validated across nine diploid human genomes; when compared to previous published results of CNVs, 40% of the insertion/deletion events were novel [7].

According to the Database of Genomic Variants, at least 25% of the human genome shows some evidence of copy number variation (hg17.v2) [8]. CNVs can account for a substantial amount of human phenotypic variability, complex traits, and disease susceptibility through the differential levels of gene expression of the involved genes [1,6]. Studies have shown associations between CNVs and phenotypic variations or disease risks including autism, schizophrenia, idiopathic learning disability, human HIV infection, systemic lupus erythematosus, inflammatory autoimmune disorders, Crohn's disease, body mass index, psoriasis, and osteoporosis etc. [2,4,9-17].

Methods and technologies for detecting genome-wide CNVs include comparative genomic hybridization to arrays, clone and PCR-product arrays, oligonucleotide arrays, and SNP genotyping arrays [18]. Among these methods, SNP genotyping arrays have shown some advantages in mapping CNVs. High-density SNP arrays offer high genomic coverage but, most importantly, allele-specific information from genotyping arrays provides an opportunity to discover CNVs in a two-dimensional level format that involves the patterns of heterozygous and homozygous genotypes available in SNP data along with the signal intensities [19]. Both PennCNV and QuantiSNP take into account the intensity values of Log R Ratio (LRR) and B allele frequency (BAF), and implement similar hidden Markov models (HMMs) [20,21]. These algorithms both assume that LRR and BAF are independent given the hidden states [20,21]. QuantiSNP implemented an objective Bayes framework. It used a resampling method to set some hyper-parameters in the priors, and applied the maximum marginal likelihood method to the training data to set other parameters [20]. In contrast, PennCNV writes the emission probabilities of LRR and BAF into the same likelihood function, and estimates the model parameters by maximizing the likelihood of observing the training data [21]. Subsequently, parameters in transition and emission probabilities are fixed in the HMM when analyzing different data. However, PennCNV provides specific parameter sets for different SNP genotyping arrays. Intuitively, in a two-dimensional space, by using both LRR and BAF, we may obtain more reliable predictions of copy number changes. In addition, we may even derive the genotype at a particular site with an abnormal copy number change [19]. Model-based two-dimensional approaches (so-called generalized genotyping approaches) have shown improved power and considerable advantages, but are more computationally demanding and require more complex algorithms to work with both sources of data [19]. All of the current algorithms call CNVs on an individual level. For example, although PennCNV provides some tools such as handling trio data or performing case-control comparison after calling CNVs on each individual, these downstream analyses have already lost power, because they do not use all the available data. PennCNV uses the Viterbi algorithm to derive a single most probable set of copy numbers for each person at each position leading to a loss of information compared to the information available from the probabilities of CNV states. For the generalized genotyping approaches, another limitation lies in the sensitivity of detecting copy number changes. At one position, the product of the transition probability of the previous state to the current state and the emission probability of being a particular state given the data needs to be relatively large to emit a copy number change, because the transition probability of being in a same state tends to be much larger than that of a change. Owing to the properties of HMM and subsequent application of the Viterbi algorithm, these methods may not be able to detect some small CNVs, and even called small CNVs may not be reliable. Finally, the Viterbi algorithm of HMM eliminates the possible case that multiple copy number states associate with the disease risk, because only the most probable chain is provided as the final result. In studies of somatic changes in tumors, for example, one can anticipate heterogeneity of states due to heterogeneity of cell populations in the tumor. In germline samples, tissues can sometimes show mosaicism, with heterogeneity of cells showing different numbers of copies of DNA in a region. In addition, the hidden CNV state may not be called with complete certainty even when normal samples are studied.

In our recent assignment of finding the missing heritability in genome-wide association studies (GWAS), CNV is one of our major concerns and needs to be further studied [22,23]. Particularly, we should pay more attention on investigating small CNVs since the distribution frequency of small CNVs (*<*1 kb) is much higher than that we expected in human genome [24]. In this research, we focused on developing a new genome-wide algorithm for SNP genotyping data to solve these problems, but this algorithm can easily be extended to other platforms if the HMM and Viterbi algorithm were implemented.

## Methods

An HMM can efficiently describe the LRR and BAF intensity data from SNP genotyping arrays. The Viterbi algorithm, which is a dynamic programming algorithm, is generally applied to reach the goal of predicting the Viterbi path, which provides the most likely sequence of hidden states representing the different copy numbers along the chromosomes. PennCNV is currently a popular CNV calling algorithm for SNP genotyping data, especially for the Illumina platform. It implements a first-order HMM that assumes that the hidden copy number state at each SNP depends only on the copy number state of the most preceding SNP [21]. It uses the Viterbi algorithm to calculate the most probable sequence of hidden states chromosome by chromosome [21]. We found in study of one dataset that PennCNV provided higher concordance on repeated assay of the same samples than did QuantiSNP overall [25] and PennCNV is an open source free software utilizing an HMM software package UMDHMM (http://www.kanungo.com/software/software.html) [26]. Six hidden states were defined in PennCNV for autosomes, corresponding to deletion of 2 copies, deletion of 1 copy, normal state, copy-neutral with loss of heterozygosity (LOH), single copy duplication, and double copy duplication or higher [21]. We developed our new genome-wide logistic regression algorithm based on the HMM implemented in PennCNV.

### Definition of the probability of being in a hidden state at a position

PennCNV calls the Viterbi algorithm to predict the most likely path of hidden copy numbers. In the Viterbi algorithm, the total probability of obtaining the path from the beginning to the current position is recorded. In every iteration, for all the jumps of any current state to any next state, the probabilities (*p*) of observing this jump are calculated, given the observed data, and are added into the total probability. For every possible next state, only the path with the largest total probability is recorded. Every *p *is calculated as the product of emission and transition probabilities at that particular position. Assuming that there are six hidden states, six paths with different ending states are recorded simultaneously. In each iteration, 36 probabilities are calculated. Each probability is a product of an emission probability and a transition probability. For every hidden state *k *at SNP *i*, six probabilities from different hidden states at SNP *i - *1 are computed. One then accumulates these six probabilities to the previous six paths. Then the one with the largest cumulative probability will be recorded as the new path with ending state *k*. Six new paths with different ending states will be forwarded to the next iteration. At the end, the Viterbi path will be selected as the one with maximum cumulative probability from the six paths obtained. From the description of the Viterbi algorithm, if we take the summation of the six probabilities from different states to ending state *k *at position *i*, we may be able to define the probability of being in state *k *at position *i*. In mathematical form, let *p_i, jk _*be the probability of the jump from state *j *to state *k *at position *i *(it is possible that *j = k*), then

where {*r_i_*, *b_i_*, *z_i_*} is the triple of LRR, BAF, and hidden copy number state at SNP *i*. On the basis of the HMM defined in PennCNV, we can define the probability of being in a hidden state *k *as

at position *i*. The probability of being in any hidden state can be defined in the same manner at that position.

One thing to notice is that the emission probability defines the probability of observing the data given a hidden state. When observations are discrete symbols, one can easily adopt discrete probability density for each hidden state in an HMM [27]. However, when observations are continuous signals, defining the emission probabilities becomes more complex. Usually a mixture of continuous probability density functions (PDFs) is used to define the emission probabilities in this situation [27]. PennCNV uses functions of normal densities in defining the emission probabilities of LRR and BAF [21]. Hence, continuous PDFs are used in defining the emission probability distributions in our case because LRR and BAF both take continuous values. And *P*(*r_i_*, *b_i_*|*z_i_*) = *P *(*r_i_*|*z_i_*) · *P *(*b_i_|z_i_*) is a product of density values. To define the probability of being in a hidden state *k *as  at position *i*, a normalization procedure is needed over all the possible hidden states since this product may not define a PDF. By simply dividing each product value by the summation of all the product values, we obtain normalized probabilities at each position, and they sum to 1.

### Logistic regression model on the defined probabilities

In PennCNV, there are six hidden states. So for each individual, at each position, we can obtain six probabilities of being in any hidden copy number state. For a large case-control study, these position-specific probabilities allow us to run a genome-wide association (GWA) test to identify CNV SNPs that are related to the disease risk. An intuitive model to fulfill the above GWA test is logistic regression. One way to reach the goal is to define a logistic regression model for each copy number. Let *p_d _*be the probability of having the disease and *C_k_*, *k *= 1, ..., 6 be the probability of being in state *k *at a particular position *i*. Then at position *i*,

By combining appropriate *C_k_*'s, we may define the probability of a deletion or duplication. For example, *C*_1 _+ *C*_2 _can be defined as the probability of a deletion at position *i*. Then the model can be written as

Similarly, the logit model for duplication can be expressed as

Note that in the logit model for deletion, only hidden states 1 and 2 that correspond to copy number 0 and 1 were involved. We did not add hidden state 3, which represents LOH in this model. One of the reasons is that the likelihood of LOH was not well defined for close markers due to linkage disequilibrium (LD) in the PennCNV HMM, so the probabilities for LOH may not be reliable. Since LOH remains a copy number neutral state, it is counted as no change from normal.

## Results

We implemented the new genome-wide algorithm in software GWCNV which is freely available online. Users should first run a modified version of PennCNV, which is included in the GWCNV package, to generate the input file that contains all the defined probabilities at each SNP. After running GWCNV, the association test results for different copy numbers at each SNP are reported in the output file specified by users. Step-by-step instructions of installing and running GWCNV are provided on the website.

Permutation tests and simulation studies were conducted to test the validity and performance of the logit algorithm, and to compare it with the association test (Fisher's exact test) based on PennCNV calls. We simply use PennCNV association test or PennCNV in the following content to describe the association test based on PennCNV copy number calls.

### Introduction to melanoma data

We tested the new algorithm using melanoma data obtained from The University of Texas MD Anderson Cancer Center. Melanoma is a malignant tumor of the melanocytes and, although not a common type of skin cancer, it accounts for 75% of all skin cancer-related deaths [28]. A total of 3,116 subjects of European continental ancestry were recruited for studies at MD Anderson Cancer Center between 1993 and 2009 in this hospital-based case-control study. This dataset included 2,053 subjects with melanoma and 1,063 subjects as age-, sex-, and ethnicity matched controls. Genotyping was performed on the Illumina HumanOmni1-Quad_v1-0_B array at the Center for Inherited Disease Research. The median call rate was 99.97%, and the error rate estimated from 69 pairs of study sample duplicates was 1*e - *5. Concordance of CNVs called by PennCNV for the sample duplicates was 73.61%. Quality control and data cleaning procedures were performed by the Gene Environment Association Studies (GENEVA) group at the University of Washington, Seattle and the Section of Computational and Genetic Epidemiology (CGE) at MD Anderson Cancer Center. Samples with gender discrepancy, unexpected related samples, non-Caucasians, outliers, etc. were identified and removed from the dataset, leaving a total of 3,021 samples. Sample data with LRR and BAF values were exported from BeadStudio software.

The melanoma data are available on the dbGaP website at the National Center for Biotechnology Information (NCBI). A large number of replicate samples, including 67 collected and 100 HapMap replicates, were involved in the genotyping process. This design allowed us to determine the quality of the data, and to compare the consistency of CNV calls by different calling algorithms. To verify the accuracy and performance of the newly developed genome-wide logistic regression algorithm, permutation tests and pseudo-simulation studies were implemented using the melanoma dataset. By randomly assigning the affection status over the samples in melanoma data, we eliminated any systematic divergence between the case and control groups. Then by comparing the distributions of the p-values from the model with the theoretical standard uniform, we were able to interpret the effectiveness and preciseness of the new algorithm. The Q-Q plots of p-value distributions for deletion and duplication are presented in the Additional File 1. No inflation of false positive rate was found in these plots for the logit algorithm and we conclude that the p-value distributions are approximately uniformly distributed.

### Simulation results

To further test the performance of the genome-wide logistic regression algorithm and to compare it with PennCNV, we designed a pseudo-simulation method. We assumed four penetrance models using a log additive model for genotype (copy number) risks. Different relative risks (RRs) for a copy number deletion ranging from 3.0 to 1.2 were embedded in these four models. We assumed that a double copy deletion causes a high risk of a disease and that a single copy deletion causes a lower risk. Let the population frequencies be *p*^2 ^and *q*^2 ^for the double copy deletion and normal copy number 2, respectively (*p *= 1 - *q*). Let *K*, *r*_0_, *r*_1_, *r*_2 _represent the population prevalence of the disease, penetrances for copy numbers 0, 1, and 2, respectively. We assumed that *p *= 0.05 and the penetrances for all other copy numbers are 0. Hence *K *can be obtained by

Under the *log *additive model, the copy number frequencies given the disease status can be derived. We then randomly selected samples to simulate on the basis of the permutation of case-control status. We used the melanoma data for our simulation studies and 2,888 samples were included. In our simulations, generally, the double copy deletion (copy number 0) represents a relatively weak signal and the single copy deletion (copy number 1) is a strong signal given the penetrance models and sample size. In fact, copy number 0 may not be very weak or copy number 1 may not be strong if the RR is high or low, respectively. In addition, for the four penetrance models, we assumed penetrances as shown in Table 1.

**Table 1:** Penetrance models for simulation

	Model 1	Model 2	Model 3	Model 4
*Pr*(*af f*|*CN *= 0)	0.09	0.0324	0.0225	0.0144
*Pr*(*af f*|*CN *= 1)	0.03	0.018	0.015	0.012
*Pr*(*af f*|*CN *= 2)	0.01	0.01	0.01	0.01

*RR*_1_	3.0	1.8	1.5	1.2

We used -5 as the theoretical LRR value for copy number 0, and -1 as the theoretical LRR value for copy number 1 to simulate the data. We chose a region on chromosome 15 that contains no CNV calls from PennCNV across all the samples. Theoretically, the data in this region are just genomic noise. By adding the theoretical values of copy number 0 and 1 onto the data, we shifted the LRR values and simulated 10 deletions with different lengths and numbers of SNPs, ranging from 3 to 58. Chromosome 15 has 34,862 SNPs in total. The 58 SNP deletion encompassed a known high LD region. By comparing the results from the logit algorithm with those from the association tests based on CNV calls from PennCNV, we could compare the sensitivity and the ability to identify different types of CNV associations of these two algorithms. Q-Q plots of *-log*_10_(*p - value*) were obtained for each penetrance model, CNV algorithm, and copy number change (0 or 1) and are presented in the Additional File 1.

When the RR was high, both algorithms performed similarly. As the RR decreased, the logit algorithm picked up signals that the PennCNV association test did not, especially when the signal was weak. When the RR was low, for both algorithms the power was too low, given the sample size, to detect such weak effects. Overall, for large CNVs, the association tests based on PennCNV calls gave more significant results (e.g., over several signals, average p-value *p *= 6.26*e *- 25 when *RR *= 3.0), but the new algorithm retained high power, especially when the disease RR was high (e.g., average p-value *p *= 3.12*e *- 11 when *RR *= 3.0). For small CNVs, however, the logistic algorithm provided smaller average p-values (e.g., *p *= 7.54*e *- 17 when *RR *= 3.0) in all the cases and can captured signals that PennCNV did not (e.g., *p *= 0.020 when *RR *= 3.0).

### Contrasting results of PennCNV versus GWCNV

**Figure 1:**
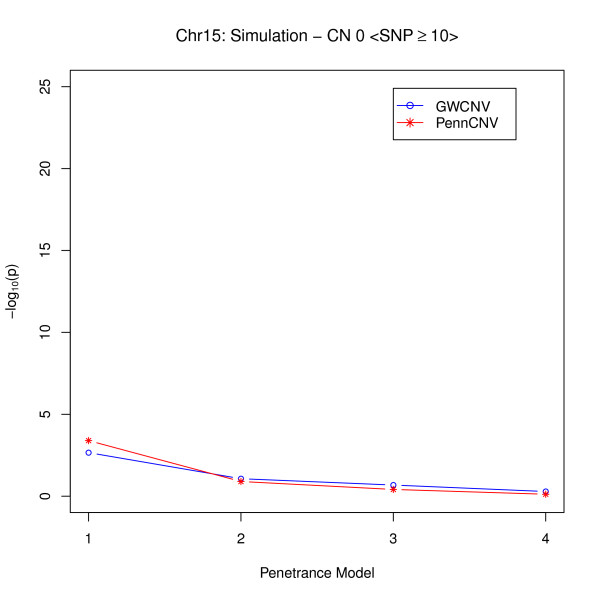
**Comparison of Logit Model and PennCNV for large CNVs (weak signal)**.

**Figure 2:**
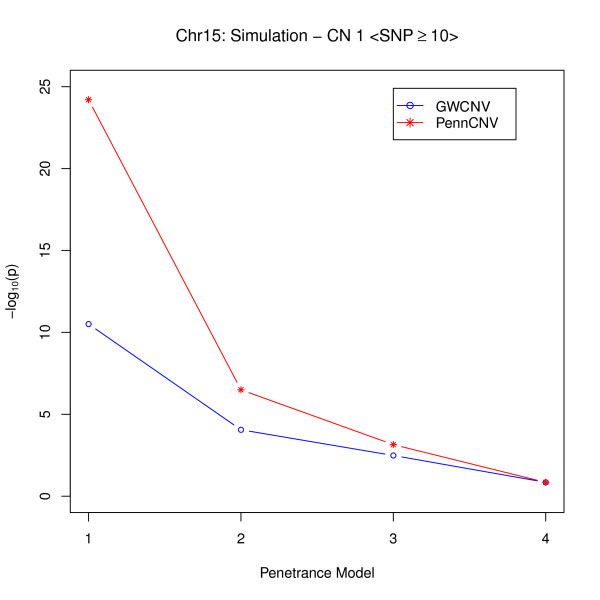
**Comparison of Logit Model and PennCNV for large CNVs (strong signal)**.

To further comprehend the difference between the logit algorithm and the PennCNV association test and to investigate the applicable conditions for both algorithms, we compared the performance of these two algorithms under different penetrance models graphically. We divided the simulated deletions into two categories: regions with at least 10 SNPs (large CNVs) and regions with fewer than 10 SNPs (small CNVs). In our simulations, five simulated CNVs fell into the first category, and the other five belonged to the second category. We calculated the average p-values for the five CNVs in each category for each copy number and penetrance model combination. We first plotted these means versus different penetrance models for copy number 0 (Figure 1) and 1 (Figure 2) when the number of SNPs was at least 10.

From Figure 1, we may conclude that when the number of SNPs is relatively large, the performance of the two algorithms is very similar if the signals are not strong. If the signals are strong, the PennCNV association test provides more significant results in different penetrance models than does the logit algorithm (Figure 2). The reason lies in the fact that for large CNVs, PennCNV may capture most of the inserted deletions. When the signals are strong, the association test applied to inferred deletions can easily provide very significant results. Whereas, the logit algorithm uses the probabilities of being in different copy numbers for the case-control comparison. In the situation of having strong signals, however, the logit algorithm is also effective because it can detect the associated CNVs.

**Figure 3:**
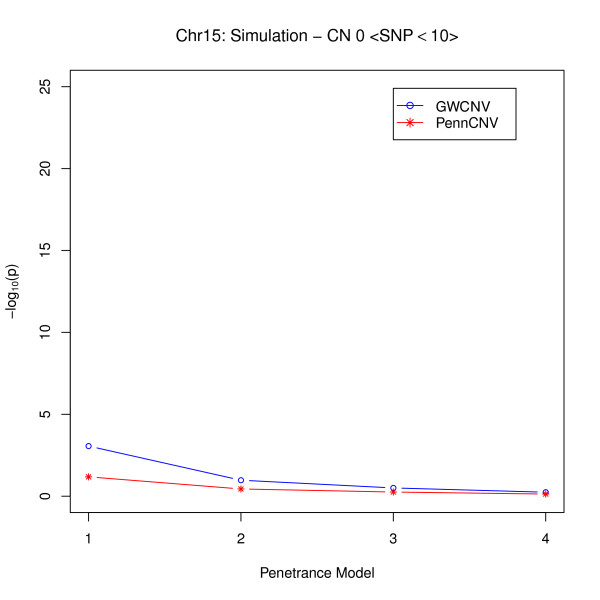
**Comparison of Logit Model and PennCNV for small CNVs (weak signal)**.

**Figure 4:**
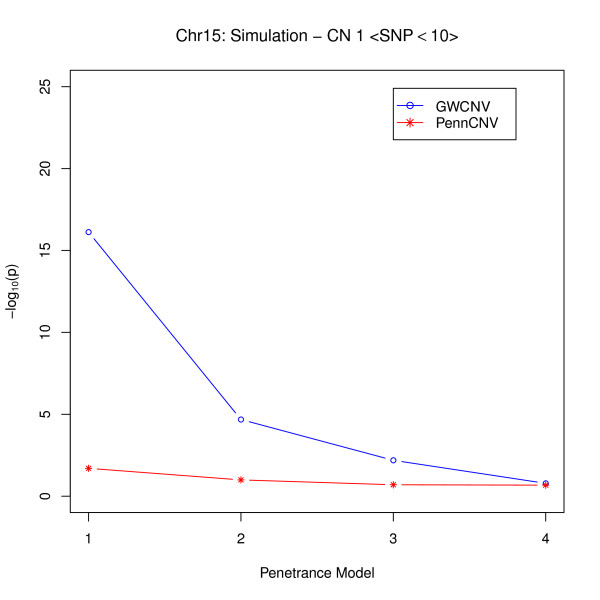
**Comparison of Logit Model and PennCNV for small CNVs (strong signal)**.

From Figure 3, we see that when the number of SNPs is relatively small, if the signals are not strong, neither algorithm provides very significant results, but the average p-values from the logit algorithm are smaller than those from the PennCNV association test. This indicates that some of the signals might only be captured by the logit algorithm since we averaged the p-values over five signals. However, for strong signals in this situation, the logit algorithm shows distinct improvements in detecting the associations between CNVs and diseases in different penetrance models (Figure 4). This implies that most signals might be caught by the logit algorithm especially when RR is high. Hence, we conclude that the logit algorithm may be more sensitive than the PennCNV association test in detecting the disease associations with small CNVs. In this simulation study, since only five signals were inserted into the data for both large and small CNV categories, power calculations may not provide reliable results (see Additional File 1).

### Power comparison and model performance

To compare the statistical power (sensitivity) and model performance of the two approaches especially for small CNVs, we conducted another simulation study using the pseudo-simulation method described above. In this study, we simulated 33 regions on chromosome 16 with 11 of them consisting of 3 SNPs, 11 of 4 SNPs, 11 of 5 SNPs. Some simulated CNVs are close to each other in this simulation. 132 SNPs were simulated in total. Assuming a significant level of 0.001, we computed the statistical power of the two methods for detecting the disease associations with 132 CNV SNPs as shown in Table 2.

**Table 2:** Power for detecting disease associations with small CNVs

		*RR*_1 _= 3.0	*RR*_1 _= 1.8	*RR*_1 _= 1.5	*RR*_1 _= 1.2
CN 0	GWCNV	0.8030	0.4773	0.3106	0.0
	PennCNV	0.2879	0.0	0.0	0.0

CN 1	GWCNV	1.0	0.9318	0.4394	0.0
	PennCNV	0.8864	0.7045	0.4015	0.0

From this table, the power of the logit algorithm is higher than at of PennCNV association test in all the scenarios, especially for weak signals and high RRs. For the strong signals (CN 1), the logit algorithm performs better than PennCNV association test under different penetrance models in terms of capturing the signals. When the signal is not strong (CN 0), the logit algorithm shows remarkable improvements. When *RR *= 1.8 and 1.5, PennCNV association test could not pick up any signal. Whereas, the logit algorithm retained power of capturing the true signals at 0.4773 and 0.3106.

**Figure 5:**
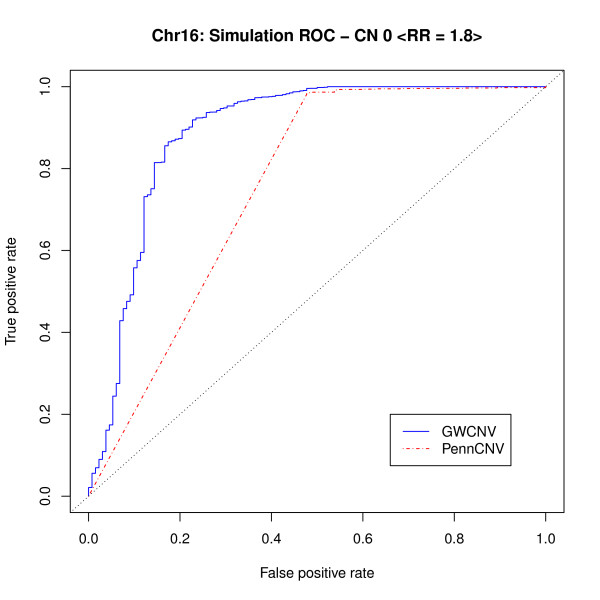
**ROC curve for simulated weak signals (*RR*_1 _= 1.8)**.

**Figure 6:**
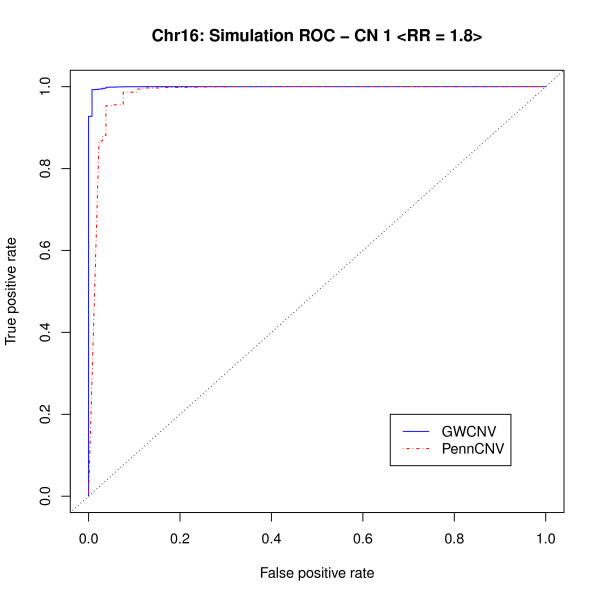
**ROC curve for simulated strong signals (*RR*_1 _= 1.8)**.

For a moderate RR 1.8 (penetrance model 2), we plotted the receiver operating characteristic (ROC) curves for the association test results from both methods to examine their performance. From both Figure 5 and 6, the logit algorithm performs well and gives better results than those from PennCNV association test. For Figure 5, the AUC for GWCNV and PennCNV association test are 0.8879 and 0.7542, respectively. For Figure 6, although the two curves are very close, PennCNV association test may take a higher false positive rate than GWCNV does under similar circumstances. Whereas, GWCNV performs almost perfectly. The false positive hits from PennCNV may due to two reasons. The transition probability for two adjacent SNPs may be small, so PennCNV may call additional SNPs after detecting a CNV. For two close CNVs, PennCNV tends to segment them into one region, so the SNPs in the middle may be called. The AUC are 0.9992 and 0.9826 for GWCNV and PennCNV association test in this case, respectively.

In general, the new genome-wide logit model is valid and performs well. It has advantages in handling the disease association tests with small CNVs. It is more powerful in detecting associations especially when the signal is not strong. It conducts association test directly on a transformation of data without calling CNVs.

## Discussion

Currently, almost all the CNV calling algorithms call CNVs at an individual level. For these algorithms, the false positive rates are high, especially for small CNVs due to the quality and properties of the intensity data, and the association tests based on called CNVs lack power in detecting CNV associations with diseases. The proposed new genome-wide logistic regression algorithm resolves the disadvantages of existing algorithms. It is a combination of an existing well-developed HMM and GWA test. It performs at a population level and controls the false positive rate well. It is more sensitive and more powerful than the existing algorithm in capturing small CNVs.

The defined probabilities of being in different hidden states reflect the statistical properties of the intensity data. By performing the association tests directly on these probabilities, power to detect the small CNVs is enhanced. The new logit algorithm uses information from all the available data, unlike existing programs such as PennCNV and QuantiSNP, which collapse much of the information into the most probable copy number states. In addition, since we apply the logistic regression model to different copy numbers at each position, the algorithm we developed could detect associations in the presence of sample heterogeneity, such as may exist in tumors and other tissues that undergo somatic changes. When there are multiple copy numbers associated with the disease at one locus, the approach to hypothesis testing we adopted may lose power since we fit tested separate models for different copy numbers. Hotelling's *T*^2^-test may be employed in this scenario for a multivariate test. However, if only a small number of "non-zero" probabilities appears in a state, one may collapse these probabilities to a neighboring state to obtain a powerful test.

Unlike PennCNV our algorithm does not include a segmentation step, and hence may more accurately reflect the underlying differences among CNV states between cases and controls. Because we do not employ segmentation and use probabilities for each state, our approach is more sensitive to small CNVs, but our method does not borrow strength from neighboring positions as much as PennCNV and hence could show weaker power for large CNVs. In the current studies we have evaluated the statistical performance of PennCNV and GWCNV for detecting duplications and deletions because we anticipate that disease risks are usually due to one of these genomic features, but it would also be possible to test for deviation from normal copy number as an alternate testing procedure.

Moreover, when the sample size is sufficient, the logistic regression model may be able to detect associations with uncommon or rare CNVs. From the results of permutation tests and simulations, we see that this algorithm may be able to pick up fine differences between cases and controls, and the false positive rate was well controlled. Employing the logistic regression model limits the use of this algorithm to case-control studies. However, adoption for quantitative traits could use an ANOVA based method.

This algorithm runs the logistic regression across the samples on each position using the probabilities of being in a hidden state. These probabilities are partially determined by the genotype states, which may be spatially correlated due to LD. LD among the genotypes would lead to a weak correlation among the test statistics. In our simulation studies we did not observe inflation of type I error rates due to this potential correlation. As one approach to evaluating any potential for bias in the test statistic, evaluating the Q-Q plot for case-control differences for deletions and duplications separately would provide insights about the behavior of the test in a particular sample. In addition, for target regions if there are concerns that strong LD could be inflating the type I error, thinning the data of SNPs in very strong LD would eliminate this concern, although it could also reduce power.

Incorporating family information into the CNV calling procedure may improve the sensitivity of detection [21]. PennCNV provides a joint calling option in its main program to detect CNVs using trio data. Studies have shown the efficacy of this strategy [29-31]. Without calling CNVs, our algorithm performs the association test directly on a transformation of the data. Essentially, case-control data are needed.

In the logit algorithm, because the association test is performed on each position, good quality control procedures may be required by this algorithm to ensure reliable results. However, sometimes the effect of factors other than the affection status is hard to eliminate in order to keep the power of the statistical tests at a desired level. In analyzing the melanoma data, we found that DNA degradation over time may strongly affect the quality of the intensity values. Furthermore, the method of obtaining the blood samples may also create unexpected divergence in the intensity values [25]. To attain valuable results from these noisy data, attention to experimental design may enhance power and reduce false positive results. In particular, using the same DNA processing procedures for cases and controls and including cases and controls on the same genotyping plates will ensure that the DNA signal intensities are comparable.

## Conclusions

In this article, we described a new genome-wide algorithm to detect CNV associations with diseases. Other than implementing a CNV calling procedure on the intensity values from SNP genotyping arrays, this algorithm performs association tests directly on the probabilities of being in a copy number at each position across case and control groups. We have proved that this algorithm is more sensitive than the existing algorithm especially when the signal is relatively weak, and it is capable of detecting associations between small CNVs and diseases.

## Authors' contributions

YX carried out the methodology and software development, and drafted the manuscript. BP participated in the design of the algorithm and provided technical support. YF provided critical comments and suggestions. CIA supervised the study design, methodology development, and helped to draft the manuscript. All authors read and approved the final manuscript.

## Supplementary Material

Additional file 1 **Supplemental results for the permutation tests and simulation studies**. Click here for file
