# Supplementary material for: Genome-wide algorithm for detecting CNV associations with diseases
Source: BMC Bioinformatics. 2011 Aug 9;12:331. doi: 10.1186/1471-2105-12-331 (PMC3173460; doi:10.1186/1471-2105-12-331)

Genome-wide algorithm for detecting CNV associations with diseases - supplemental results

Yaji Xu[1,2] , Bo Peng[1] , Yunxin Fu[2] & Christopher I. Amos[1*]

[1]Department of Epidemiology, The University of Texas MD Anderson Cancer Center, 1155 Pressler St., Houston, Texas 77030, USA
[2]Division of Biostatistics, The University of Texas School of Public Health, 1200 Pressler St., Houston, Texas 77030, USA

Email: Yaji Xu - yajixu@mdanderson.org, yajixu@gmail.com; Bo Peng - bpeng@mdanderson.org; Yunxin Fu - yunxin.fu@uth.tmc.edu; Christopher I. Amos*- camos@mdanderson.org;

*Corresponding author

Figure 1: Q-Q plot of $-log_{10}(p-value)$ from Logit Model permutation tests for deletion on chromosome 15

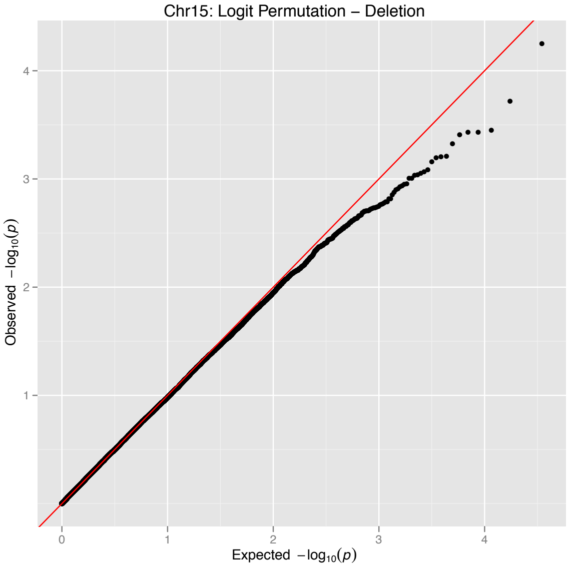

Supplemental results

Permutation tests for GWCNV

We ran the logit algorithm on the melanoma data based on a random permutation of the case-control status to test the false positive rate of the new algorithm. The random permutation of case-control status was generated in R software. The Q-Q plots show the distributions of p-values from the logit algorithm versus the theoretical standard uniform distribution for deletion (Figure 1) and duplication (Figure 2) on chromosome 15.

In Figure 1, the fact that most of the points lie on the diagonal indicates that the p-values from the logit algorithm distribute approximately uniformly on [0, 1]. Although some points are under the diagonal when the $-log_{10}(p-value)$ is high, the percentage of those points versus the total amount of points is low due to the property of uniform distribution. An appropriate measure to visualize the distribution of large amount of p-values is the inflation factor λ. For the permutation tests for deletion, $\lambda = 1.0052$. This result suggests the conclusion of no inflation of false positive rate. We may conclude that the p-value distribution for deletion is approximately a standard uniform.

In the duplication case of the permutation tests, the p-values from the logit algorithm distribute as a standard uniform properly in Figure 2. The inflation factor $\lambda = 1.0264$ in this case.

Figure 2: Q-Q plot of $-log_{10}(p-value)$ from Logit Model permutation tests for duplication on chromosome 15

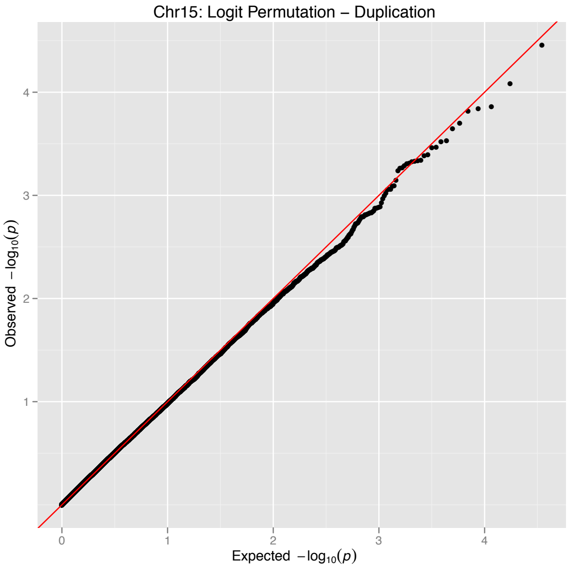

Permutation tests for association test applied to PennCNV calls

Association tests based on PennCNV calls were also run using the permutation of case-control labels. The Q-Q plots in Figures 3 and 4 show the distributions of p-values from the PennCNV association test versus the theoretical standard uniform distribution for deletion and duplication on chromosome 15, respectively. The Q-Q plots for the PennCNV association test show skewed distributions compared with the standard uniform distribution. Note that we deleted the results for which there were few cases or controls with copy number change. These plots indicate that this test yielded an excess of p-values with inflated values, suggesting this test may have low power.

Simulation results on chromosome 15

In penetrance model 1, $RR = 3.0$ and disease prevalence $K = 0.0121$. On the basis of the assumed copy number frequencies, we obtained relatively strong signals for both copy number 0 and 1.

From Figures 5 and 6, both algorithms were able to capture the signals of the double copy deletion. Note that the scales of X- and Y-axes are different between the Q-Q plots for the logit algorithm and the PennCNV association test. Also, after removing the non-informative points with 0 counts for copy number 0 from both cases and controls, there is a relatively small number of points in the Q-Q plot for PennCNV. Hence, Figure 6 may not reflect well the real performance of the PennCNV association test, which could

Figure 3: Q-Q plot of $-log_{10}(p-value)$ from the PennCNV permutation tests for deletion on chromosome 15

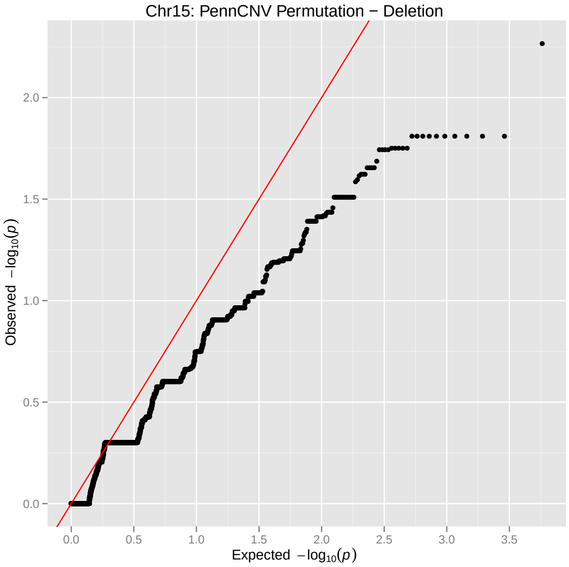

Figure 4: Q-Q plot of $-log_{10}(p-value)$ from the PennCNV permutation tests for duplication on chromosome 15

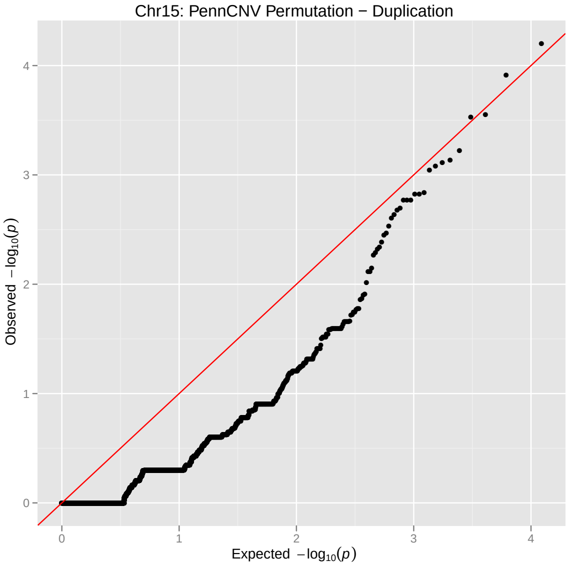

Figure 5: Q-Q plot of $-log_{10}(p - value)$ from Logit Model simulation for copy number 0 on chromosome 15 ($RR_1 = 3.0$)

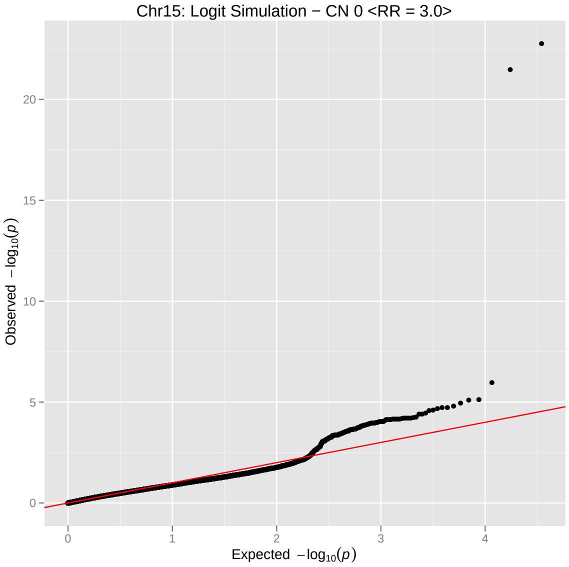

Figure 6: Q-Q plot of $-log_{10}(p - value)$ from PennCNV simulation for copy number 0 on chromosome 15 ($RR_1 = 3.0$)

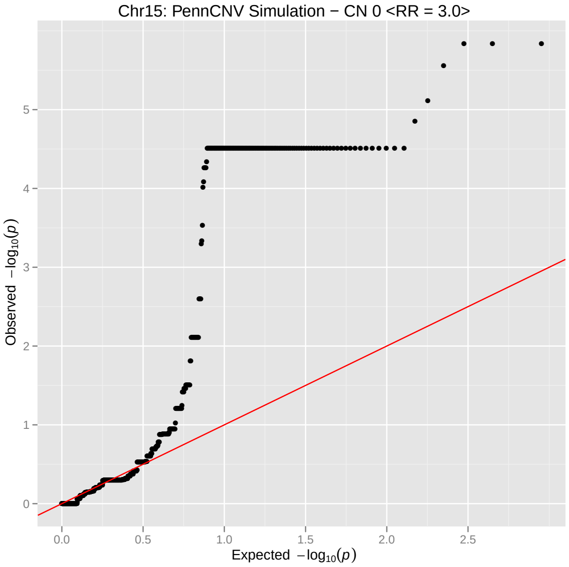

Figure 7: Q-Q plot of $-log_{10}(p - value)$ from Logit Model simulation for copy number 1 on chromosome 15 ($RR_1 = 3.0$)

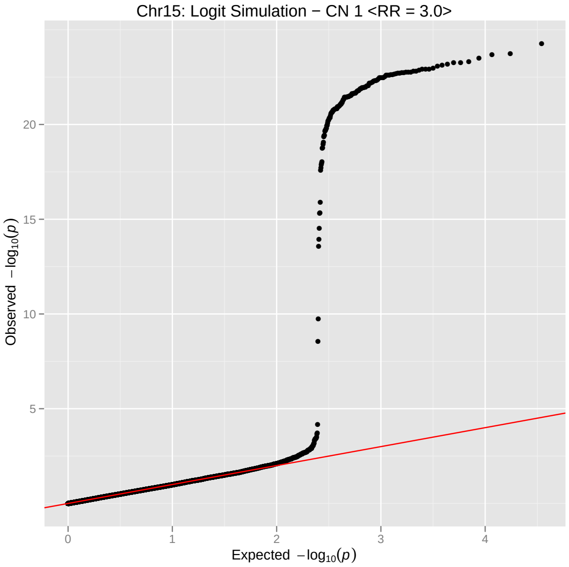

have lower power than predicted. If we put the non-informative points back into the graph, the Q-Q plot will move horizontally to the right due to a large amount of 0s. The Q-Q plot of the logit algorithm (Figure 5) behaves satisfactorily. The most significant two positions are in the simulated regions. Because PennCNV implemented a segmentation procedure after the calling procedure by the Viterbi algorithm, p-values within the same CNV region take the same numerical value.

Figures 7 and 8 show the situation results of single copy deletion under penetrance model 1. We see that both algorithms provide similar results. For copy number 1, we generated very strong signals. PennCNV got lower p-values in some parts of its distribution due to the strength of the signals (Figure 8). In this case, PennCNV may be able to identify all the individuals with the simulated CNV region, hence generating more significant p-values, whereas the logistic regression algorithm uses probabilities to detect the associations.

Plots in Figures 9, 10, 11, and 12 show results for the penetrance model 2, in which $RR = 1.8$ and $K = 0.010816$. Under penetrance model 2, the simulated signals for copy number 0 became weak, and it becomes hard for both algorithms to detect them with confidence (Figures 9 and 10).

For copy number 1, the moderate signals show up on both Q-Q plots for the logit algorithm and the PennCNV association test (Figures 11 and 12, respectively). Both algorithms behave well, but PennCNV shows a small region that is skewed to the right compared with the standard uniform distribution (see

Figure 8: Q-Q plot of $-log_{10}(p - value)$ from PennCNV simulation for copy number 1 on chromosome 15 $(RR_1 = 3.0)$

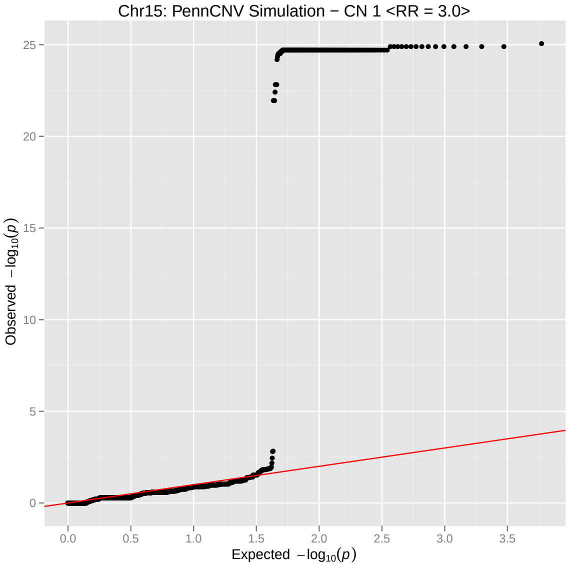

Figure 9: Q-Q plot of $-log_{10}(p - value)$ from Logit Model simulation for copy number 0 on chromosome 15 $(RR_1 = 1.8)$

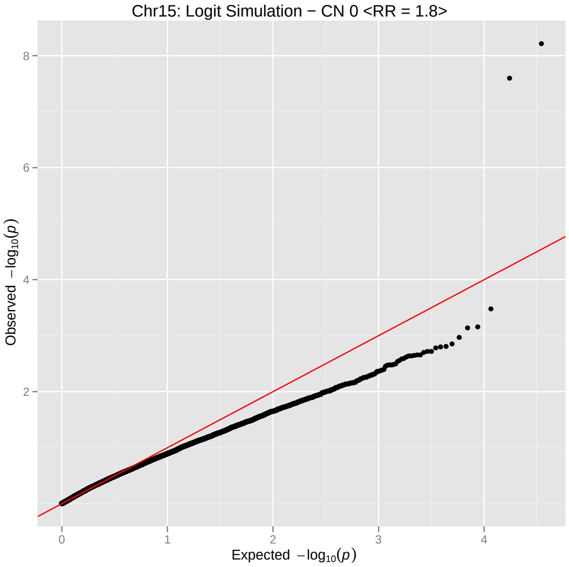

Figure 10: Q-Q plot of $-log_{10}(p - value)$ from PennCNV simulation for copy number 0 on chromosome 15 $(RR_1 = 1.8)$

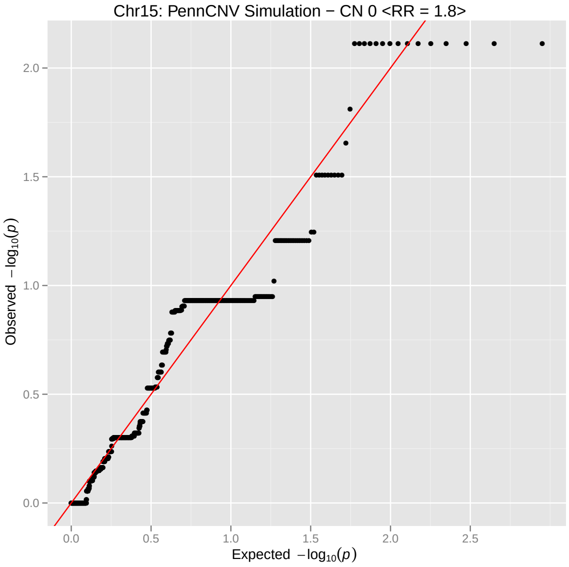

Figure 11: Q-Q plot of $-log_{10}(p - value)$ from Logit Model simulation for copy number 1 on chromosome 15 $(RR_1 = 1.8)$

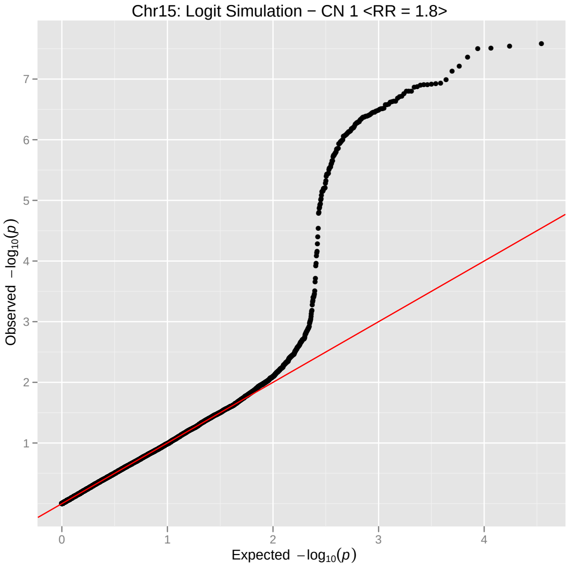

Figure 12: Q-Q plot of $-log_{10}(p - value)$ from PennCNV simulation for copy number 1 on chromosome 15 ($RR_1 = 1.8$)

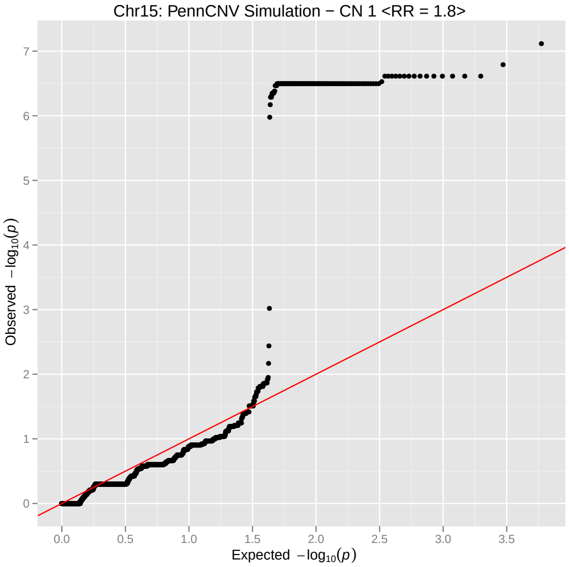

Figure 12).

For penetrance models 3 and 4, the signals became weak, and the power of the two algorithms is too low given the sample size to detect such weak effects.

Power comparison for simulations on chromosome 15

Table 1 - Power for detecting disease associations with large CNVs

		$RR_1 = 3.0$	$RR_1 = 1.8$	$RR_1 = 1.5$	$RR_1 = 1.2$
CN 0	GWCNV	0.3017	0.0172	0.0	0.0
	PennCNV	0.9741	0.0	0.0	0.0
CN 1	GWCNV	1.0	0.9655	0.0259	0.0
	PennCNV	1.0	1.0	0.0	0.0

Table 2 - Power for detecting disease associations with small CNVs

		$RR_1 = 3.0$	$RR_1 = 1.8$	$RR_1 = 1.5$	$RR_1 = 1.2$
CN 0	GWCNV	0.0833	0.0	0.0	0.0
	PennCNV	0.3333	0.0	0.0	0.0
CN 1	GWCNV	1.0	0.9167	0.0417	0.0
	PennCNV	0.875	0.875	0.0	0.0

Assuming a significant level of 0.0001, we computed the statistical power of the logit algorithm and PennCNV association test for detecting the disease associations in the first simulation study on chromosome 15. The results are shown in Table 1 and 2.

In this simulation study, since there are only five signals for each CNV category (large or small), the results from power calculations may not be reliable. From Table 1, overall the PennCNV association test performs better than GWCNV for large CNVs, though for some cases GWCNV shows higher power. For example, for copy number 1, when $RR = 1.5$, power from GWCNV is 0.0259, whereas it is 0 for PennCNV association test. For small CNVs, GWCNV surpasses PennCNV association test in performance. Tough it is contrary for the case of copy number 0 when $RR = 3.0$, if we loose the significant level to 0.001, the numbers will change to 0.8333 and 0.3333 for GWCNV and PennCNV association test, respectively.

PennCNV calling error rate

Table 3 - PennCNV false negative calling rate on the simulated chromosome 15 data

	Large CNVs	Small CNVs
CN 0	6.76%	49.41%
CN 1	6.05%	28.59%

Using the PennCNV calling results on the simulated chromosome 15 data, we calculated the error rates of this algorithm. A call is counted as a true positive if it is within one of the simulated regions. From the results in Table 3, the false negative rates are low when the CNVs are large. For small CNVs, the false negative calling rates are high, especially for the CNVs with relatively low frequencies (49.41% for copy number 0). We may also calculate an upper bound of the false positive rate by assuming that there is no CNV outside the simulated regions. The upper bounds of the false positive rates for copy number 0 and 1 are 48.87% and 51.87%, respectively.

Additional ROC curves for simulations on chromosome 16

For penetrance model 1 ($RR_1 = 3.0$), we plotted the receiver operating characteristic (ROC) curves for the association test results from both methods. In these plots, the colorized line on the right represents different cutoffs. From both Figure 13 and Figure 14, the logit algorithm provides better results than those from PennCNV association test. For Figure 13, the AUC for GWCNV and PennCNV association test are 0.9652 and 0.8666, respectively. For Figure 14, two curves are very close. The AUC are 1.0 and 0.9958 for GWCNV and PennCNV association test in this case, respectively. Two methods show closer performance than that from penetrance model 2 since the RR is high in this scenario.

Figure 13: ROC curve for simulated weak signals ($RR_1 = 3.0$)

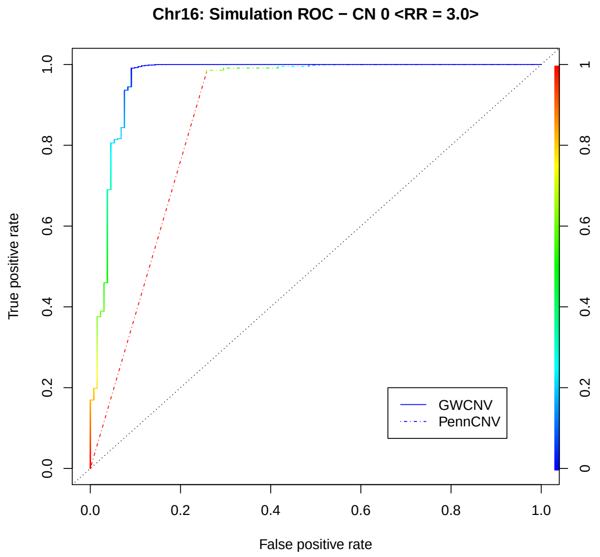

Figure 14: ROC curve for simulated strong signals ($RR_1 = 3.0$)

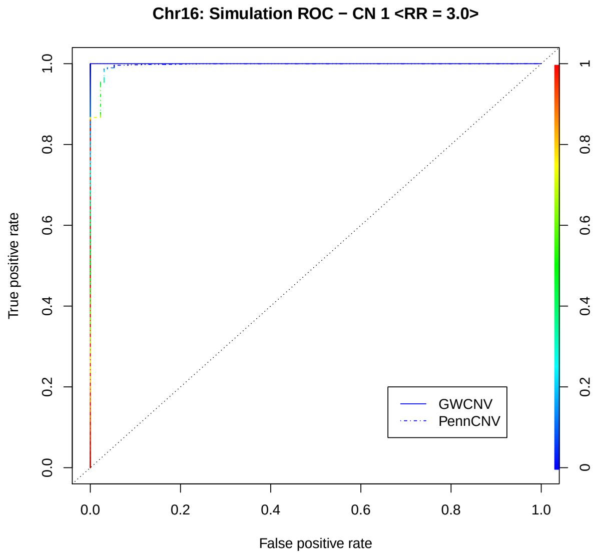

Supplement: Additional file 1 — Supplemental results for the permutation tests and simulation studies. [file 1471-2105-12-331-S1.PDF]
